# NXPH4 can be used as a biomarker for pan-cancer and promotes colon cancer progression

**DOI:** 10.18632/aging.205648

**Published:** 2024-04-12

**Authors:** Zhipeng Zhang, Pengfei Wang, Siwen Chen, Dezhi Xiang, Jinzhen Chen, Wanchang Huang, Xiao Liu, Tongwen Yi, Dawei Wang, Yunfei Pu, Longfu He, Hao Zhang

**Affiliations:** 1 Department of Anorectal Surgery, The Fifth Affiliated Hospital of Zunyi Medical University, Zhuhai, China; 2 Department of Gastrointestinal Surgery, Xijing Hospital, Fourth Military Medical University, Xi’an, China; 3 The Sixth Affiliated Hospital of Harbin Medical University, Harbin, China; 4 Key Laboratory of Hepatosplenic Surgery, The First Affiliated Hospital of Harbin Medical University, Harbin, China; 5 Department of Physiology, Zhuhai Campus of Zunyi Medical University, Zhuhai, China

**Keywords:** NXPH4, colon cancer, pan-cancer, biomarker, bioinformatics

## Abstract

NXPH4 promotes cancer proliferation and invasion. However, its specific role and mechanism in cancer remain unclear. Transcriptome and clinical data for pan-cancer were derived from the TCGA database. K-M survival curve and univariate Cox were used for prognostic analysis. CIBERSORT and TIMER algorithms were employed to calculate immune cell infiltration. Gene set enrichment analysis (GSEA) was employed for investigating the function of NXPH4. Western blot verified differential expression of NXPH4 in colon cancer. Functional assays (CCK-8, plate clonogenicity assay, wound healing assay, and Transwell assay) confirmed the impact of NXPH4 on proliferation, invasion, and migration of colon cancer cells. Dysregulation of NXPH4 in pan-cancer suggests its potential as a diagnostic and prognostic marker for certain cancers, including colon and liver cancer. High expression of NXPH4 in pan-cancer might be associated with the increase in copy number and hypomethylation. NXPH4 expression in pan-cancer is substantially linked to immune cell infiltration in the immune microenvironment. NXPH4 expression is associated with the susceptibility to immunotherapy and chemotherapy. Western blot further confirmed the higher expression of NXPH4 in colon cancer. Knockdown of NXPH4 significantly suppresses proliferation, invasion, and migration of colon cancer cell lines HT-29 and HCT116, as validated by functional assays.

## INTRODUCTION

The incidence of cancer has gradually increased since the start of the twenty-first century. As a result, it has grown to be a significant factor in both quality of life and life expectancy of people. It is primarily caused by genetic and environmental influences [1, 2]. Malignancies have a complex microenvironment of malignant cells, which makes it difficult to eradicate them [3]. Moreover, several reports have highlighted that the phenotype of malignancies exhibits a strong correlation with the tumor immune microenvironment [4]. The International Agency for Research on Cancer (IARC) of the World Health Organization reported over 19 million new cancer cases and nearly 10 million deaths from cancer in the world last year alone [1]. It is generally accepted that if a country has a relatively high life expectancy, a high level of education, and a high standard of living, it tends to have a higher incidence of cancer [5]. Surgery, radiotherapy, and chemotherapy are the traditional means of cancer treatment in clinical practice [6, 7]. However, the efficacy of the traditional means of treatment does not currently meet expectations, and the emerging field of immunotherapy has emerged as a promising cancer treatment [8, 9]. This is especially true of immunotherapies that prolong the expected survival of the patient [10]. However, the sensitivity of immunotherapy is currently low and predictive markers are critical for immunotherapy. In the last decade, with the development of bioinformatics, pan-cancer analysis of some oncogenes or tumor suppressor genes can be performed, discovering the prevalent mechanisms of tumourigenesis and providing potential targets and practical approaches for future cancer treatment [11].

Neurexophilin4 (NXPH4) is a neuro synaptic secretory protein. It is an essential neuropeptide-like glycoprotein that belongs to the neurophilic rexophilin family [12]. NXPH4 promotes cancer cell proliferation, migration, and invasion and is vital in the tumor metastasis cascade [12]. Up to now, members of this family have been identified, including NXPH1, NXPH2, NXPH3, and NXPH4, all of which share a common structure of five domains [13, 14]. However, very little research has been done on the function of NXPH4 in recent decades [15, 16]. Recent studies suggest that NXPH4 may function as a crucial gene in MIBC development and immune regulation [17]. Tissue validation results have demonstrated a higher expression level of NXPH4 in cancers such as cervical squamous cell carcinoma, endocervical squamous cell carcinoma, cholangiocarcinoma, invasive breast cancer, colorectal adenocarcinoma, and oesophageal cancer [18]. Moreover, NXPH4 can be used as a molecular target to modulate the effects of immunotherapy [12]. In addition to its ability to promote tumourigenesis, NXPH4 may also be significant in tumor immunomodulation [17]. In some types of cancer, the expression of NXPH4 did not differ significantly between cancerous and normal tissues. This suggests that NXPH4 may be an oncogene in several types of tumors and may prevent tumor cell genesis in other types of tumors. Additionally, the upregulation and downregulation of NXPH4 in tumor cells also promote and inhibit cell proliferation, respectively [12]. Furthermore, high NXPH4 expression also promotes resistance to gemcitabine [12]. In the diagnosis of hepatocellular carcinoma, NXPH4 can also significantly improve the diagnostic rate as well as early diagnosis [18]. Therefore, further experiments were conducted, which aimed at exploring the NXPH4 expression levels in other cancers and the potential mechanisms of high expression using bioinformatics.

This study comprehensively analyzed the abnormal expression, copy number variation, methylation, and prognostic value of NXPH4 in pan-cancer, including a combined comparative analysis of 33 cancers downloaded from TCGA and GTEx databases. Moreover, *in vitro* studies confirmed the oncogenic effect of NXPH4 in colon cancer.

## MATERIALS AND METHODS

### Data source

Normal tissue data from the TCGA and GTEx databases, as well as tumor data for 33 different cancer types, were retrieved from XENA (https://xenabrowser.net/datapages/).

### Survival analysis

The prognostic indicators used in the current research involved overall survival (OS), disease-specific survival (DSS), and progression-free survival (PFS). Univariate Cox analysis and Kaplan-Meier (K-M) survival analysis were carried out to analyze the association of NXPH4 with prognosis. For KM survival analysis, NXPH4 was categorized into high- and low-expression groups based on the median mRNA levels, and a comparison of the survival differences between the two groups was made.

### Immune infiltration analysis

To reliably assess immune correlations, the study used immunedeconv, an R package incorporating six state-of-the-art algorithms, such as TIMER, xCell, MCP-counter, CIBERSORT, EPIC, and quanTIseq. Using pan-cancer expression profile data, CIBERSORT and TIMER were used to assess immune cell infiltration levels in each cancer sample and correlate them with NXPH4 expression [19]. In addition, the TIGER database was utilized to investigate NXPH4 expression in each subtype of colorectal cancer cells (http://tiger.canceromics.org/#/) [20].

### Tumor mutation burden (TMB) and microsatellite instability (MSI)

TMB was derived from The Immune Landscape of Cancer, articled by Thorsson et al. in 2018 [21]. In addition, MSI was retrieved from Landscape of Microsatellite Instability Across 39 Cancer Types, published by Bonneville et al. in 2017 [22]. Furthermore, the NXPH4 levels were correlated with TMB and MSI values, and the results were presented using radar plots.

### Drug sensitivity

TIDE is an algorithm that calculates the sensitivity of patients to immunotherapy based on mRNA expression in tumor samples [23]. TIDE has been used to investigate sensitivity to immunotherapy in colon adenocarcinoma (COAD) patients in the TCGA database, with higher TIDE scores associated with less effective immunotherapy and shorter survival following immunotherapy [24]. The link between the NXPH4 levels and the IC50 of chemotherapeutic drugs was assessed by utilizing the Gene Set Cancer Analysis (GSCA) database.

### Protein-protein interaction (PPI) network

GeneMANIA (http://genemania.org) is an easily accessible online database that enables researchers to examine the role and interactions among particular genes or gene sets of interest [25]. The current research looked for proteins that interact with members of the ANP32 family in humans and established PPI networks using GeneMANIA.

### Functional enrichment analysis

CancerSEA (http://biocc.hrbmu.edu.cn/CancerSEA/) aims to investigate various functional states of cancer cells at the single-cell level in a comprehensive manner and offers a functional state map of single cancer cells from 41,900 single cancer cells across 25 cancer types [26]. The current research utilized CancerSEA to assess the functional relevance of NXPH4 in cancer.

For gene set enrichment analysis (GSEA), the gene sets “c2 KEGG”, “c2 Wiki pathway,” and “c2 REACTOME” gmt files were initially retrieved from the MSigDB database (https://www.gsea-msigdb.org/gsea/msigdb/index.jsp). GSEA based on c2 KEGG, c2 WIki, and c2 REACTOME was then performed by employing the clusterProfiler package of the R software (significant enrichment was considered at p-value < 0.05 and q-value < 0.25).

### Cell culture

Human colorectal cancer cell lines HT29 and HCT116 were provided by iCell Bioscience Inc. (Shanghai, China). McCoy’s 5A medium (Procell, China) containing 10% fetal bovine serum (FBS; Gibco, Carlsbad, CA, USA) was used for culturing the above cell lines, followed by incubation of the cells at 37° C, in the presence of 5% CO_2_.

### Western blot

The cells were frozen with cold radioimmunoprecipitation (RIPA) lysis buffer, which contained 1% protease inhibitor, for 30 minutes on ice. Subsequently, they were subjected to centrifugation at 13,500 rpm for 15 minutes at a temperature of 4° C. The supernatants, which contained the total protein content, were collected, and the CBA method was employed to measure the protein concentration. Separation of the same quantity of total protein was achieved by SDS-PAGE, using an 8% to 12% polyacrylamide gel method, followed by transferring it onto a PVDF membrane. Blocking of the membranes was done with 5% skimmed milk for 2 hours, followed by incubation with primary antibodies NXPH4 (Abcam, ab74999, Cambridge, MA, USA) at 4° C overnight while shaking. Following PBST elution, the fluorescent secondary antibody (LI-COR Biosciences, Lincoln, USA) was added to the sample and incubated at room temperature for 1 hour. The variations in the related proteins in individual groups were scanned with the aid of an Odyssey CLx Imaging System (LI-COR Biosciences, Lincoln, NE, USA), with GAPDH serving as the internal control. Finally, Image Studio Software was employed for analyzing and processing the protein bands.

### RT-qPCR

The total RNA extraction from the cells was done by utilizing an RNA extraction kit (Axygen Scientific lnc, Silicon Valley, CA, USA). cDNA synthesis was done by employing a Toyobo Reverse Transcription Kit, and SYBR GREEN reagent was employed for detecting the expression of the corresponding gene. GAPDH was utilized as an internal reference gene. Moreover, the 2^-ΔΔCt^ method was employed to examine the data. NXPH4 forward primer: AAGGTCTTCGGACGGCCTA; reverse primer: GCAGCGAAAACTTGAGGGTAT. GAPDH forward primer: CTGGGCTACACTGAGCACC; reverse primer: AAGTGGTCGTTGAGGGCAATG.

### Cell transfection

A single-cell suspension was prepared by utilizing cells from the logarithmic growth phase, and the cells were then seeded onto a plate and subjected to growing for 24 hours. Once the cells reached approximately 30% confluence, the lentivirus sh-NXPH4 (Genechem, Shanghai, China), which had been prepared and packaged, was added to the HT29 and HCT116 cell culture medium following the provided guidelines. The cells were then cultured for 10 to 12 hours at an MOI of 20. Following transfection, the stably transfected strain was selected using puromycin (HT29 puromycin concentration was 4ug/ml, while HCT116 puromycin concentration was 2ug/ml). The transfection efficiency was determined by means of RT-qPCR and Western blot.

### CCK-8

Cell proliferation was measured by CCK-8 assays. Cells (1 × 10^3^ cells/well) were plated in the medium in 96-well plates (200 μL). After 24 hours, the medium was replaced with a mixture of CCK8 and cell culture medium (110 μL, 1:100) after wrapping in tin foil, followed by incubation for 2 hours. OD values were calculated at 490 nm by a microplate reader. The normal cell medium was changed at the end of the experiment.

### Plate clonogenicity assay

Briefly, HT29 and HCT116 cells were transfected by sh-NC or sh-NXPH4 and seeded in 6-well plates (1 × 10^3^ cells/well). The cells were subjected to growing in a culture medium with 10% FBS. The cell culture was maintained at a temperature of 37° C and in the presence of 5% CO_2_ for a duration of 14 days. Afterward, the cell colonies were fixed using methanol, subjected to staining with 0.5% crystal violet, and counted manually.

### Wound healing assay

Cell migration was assessed by conducting a wound-healing assay. Cell culturing was done in 6-well plates until they were completely confluent. Subsequently, a scratch was created in the cell monolayer using a plastic pipette tip measuring 100 μL. The cells were then washed and kept in a medium devoid of serum. The scratch width was recorded under a microscope (Olympus Corporation, Tokyo, Japan) at 0 hours and 24 hours after scratching. Cell migration was quantified in multiple regions and presented as the rate of closure.

### Transwell assay

Matrigel and serum-free medium were added to the membrane in the upper Transwell chamber, which was air-dried for 2 hours at 37° C. Subsequently, a serum-free cell suspension (4 × 10^4^ cells), measuring 500 μL, was introduced into the upper chamber. The lower chamber was added with 800 μL of medium containing 20% FBS. It was then placed in an incubator and subjected to incubation for a duration of 24 hours. Following the incubation period, the cells residing on the membranes were immobilized by treating them with 4% paraformaldehyde at room temperature for a duration of 20 minutes. Subsequently, the fixed cells were subjected to staining with crystal violet, which lasted for 15 minutes. Finally, images of the samples were captured by utilizing an inverted microscope (Olympus, 400x magnification). The migrated cell count was counted by employing the Image J software.

### Statistical analysis

The selection of statistical tests for comparing continuous variables between two groups of data depended on the distribution of the data. If the data followed a normal distribution, either the t-test or the Mann-Whitney U-test was employed. On the other hand, when comparing continuous variables among three groups of samples, the Kruskal-Wallis test was utilized. For categorical data, the chi-square test was employed. Lastly, all correlations were examined by means of Spearman correlation analysis. Furthermore, survival analysis was carried out by employing the Kaplan-Meier method and tested using the log-rank method. Statistical analyses were conducted by utilizing the R software, and the significance level was established at a threshold of *P* < 0.05 (*, *P* < 0.05; **, *P* < 0.01; ***, *P* < 0.001).

## RESULTS

### NXPH4 is upregulated in pan-cancer and can be used as a diagnostic marker

**Figure 1:**
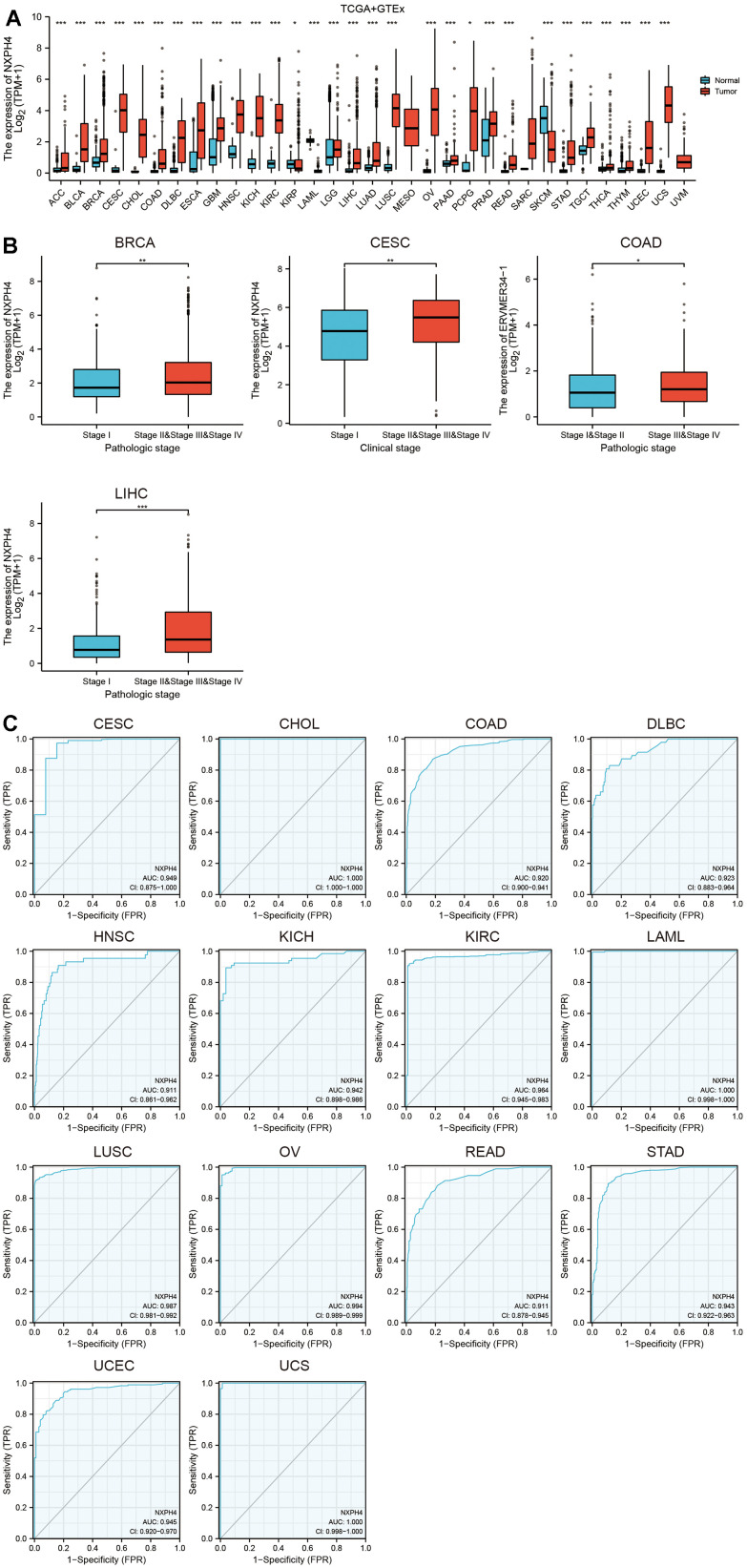
**Abnormal expression and diagnostic ability of NXPH4 in pan-cancer.** (**A**) Differential expression of NXPH4 between pan-cancer and corresponding normal tissues in TCGA combined GTEx database. (**B**) Differential expression of NXPH4 in early and late cancer tissues of BRCA, CESC, COAD, and LIHC. (**C**) Diagnostic ROC curve of NXPH4 in pan-cancer.

The study highlighted a higher expression of NXPH4 in almost all cancers except Kidney renal papillary cell carcinoma (KIRP), Acute Myeloid Leukemia (LAML), Skin Cutaneous Melanoma (SKCM), Thyroid carcinoma (THCA), and thymoma (THYM) (Figure 1A). NXPH4 levels were observed in the initial stages of Breast invasive carcinoma (BRCA), Cervical squamous cell carcinoma and cervical adenoma (CESC), COAD, and Liver hepatocellular carcinoma (LIHC), and its expression increased significantly as pathological and clinical stages progressed (Figure 1B). (Figure 1C, all AUC > 0.9), indicating its role as a potential biomarker for many cancers.

### Copy number variation and methylation of NXPH4

**Figure 2:**
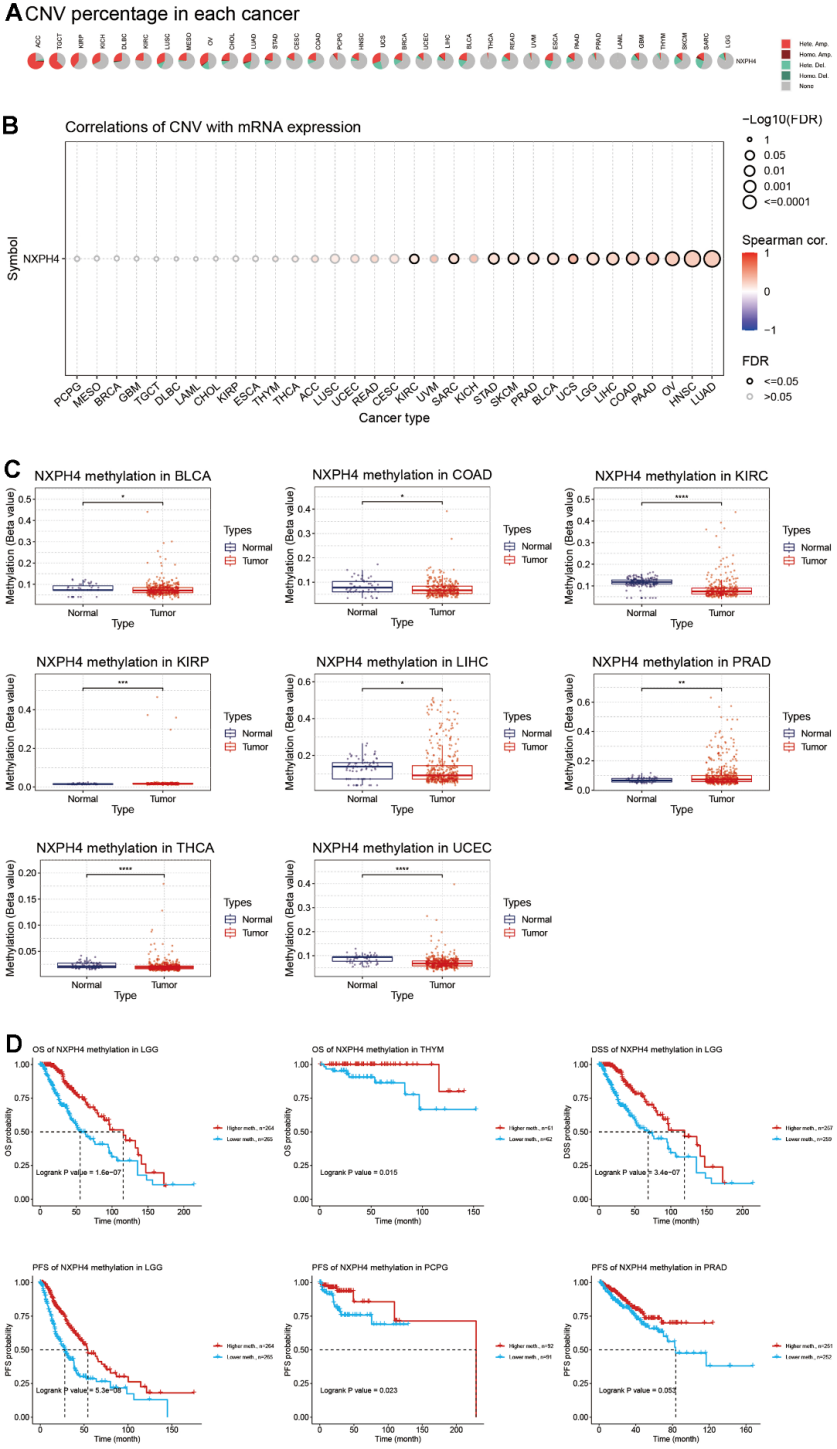
**Copy number variation and methylation of NXPH4 in pan-cancer.** (**A**) Copy number variation in NXPH4 in pan-cancer. (**B**) Correlation between copy number variation and mRNA expression level of NXPH4 in pan-cancer. (**C**) Differential methylation levels of NXPH4 between pan-cancers and corresponding paracancer tissues. (**D**) Relationship between NXPH4 methylation levels and prognosis in pan-cancers.

To explore the mechanisms by which NXPH4 may be dysregulated, the study examined its copy number variation and methylation levels. Figure 2A shows the copy number variation of NXPH4 in pan-cancer. NXPH4 was differentially deleted and amplified in various cancers, including Adrenocortical carcinoma (ACC), Testicular Germ Cell Tumors (TGCT), KIRP, KICH, DLBC, KIRC, LUSC, Mesothelioma (MESO), OV, CHOL, Lung adenocarcinoma (LUAD), STAD, CESC, COAD, Pheochromocytoma and Paraganglioma (PCPG), HNSC, UCS, BRCA, UCEC, LIHC, and Bladder Urothelial carcinoma (BLCA). In THCA, READ, Uveal Melanoma (UVM), Esophageal carcinoma (ESCA), and Pancreatic adenocarcinoma (PAAD), amplification mutations predominated, but deletion mutations were still present to varying degrees (Figure 2A, 2B). The methylation levels of NXPH4 in BLCA, KIRC, KIRP, LIHC, PRAD, THCA, and UCEC were lower than in the corresponding paracancerous tissue (Figure 2C), indicating that the upregulation of NXPH4 in these cancers might be caused by hypomethylation. Further analysis highlighted that increased NXPH4 methylation had a link to improved OS, DSS, and PFS (Figure 2D). In THYM, a link between higher levels of NXPH4 methylation and improved overall survival (OS) was observed (Figure 2D). Similarly, in PCPG and PRAD, higher levels of NXPH4 methylation were linked to better progression-free survival (PFS) (Figure 2D).

### Prognostic value of NXPH4 in cancers

**Figure 3:**
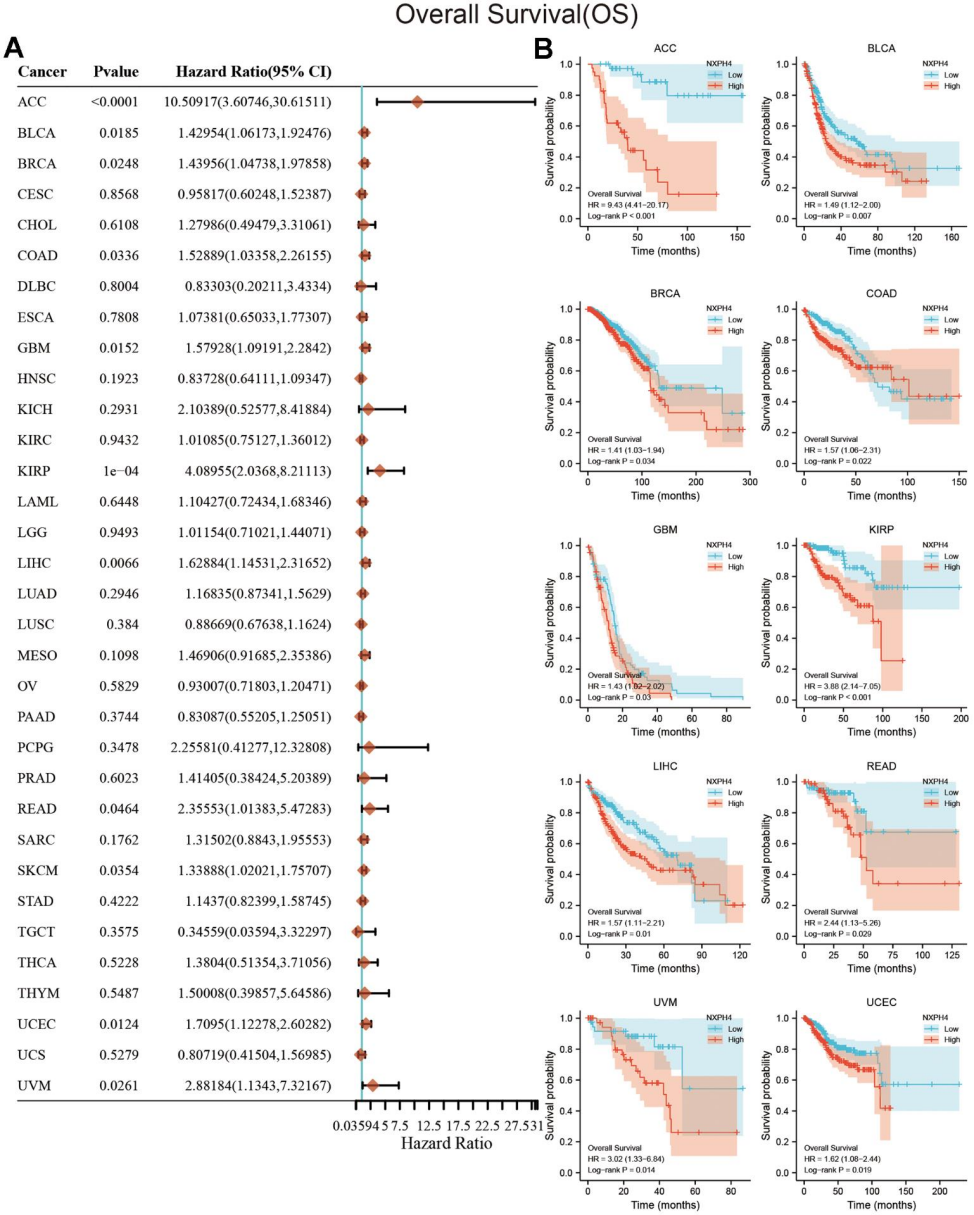
**Relationship between NXPH4 expression and OS in pan-cancer.** (**A**) Univariate Cox analysis showed the relationship between NXPH4 and OS in pan-cancer. (**B**) K-M survival analysis showed the relationship between NXPH4 and OS in pan-cancer.

**Figure 4:**
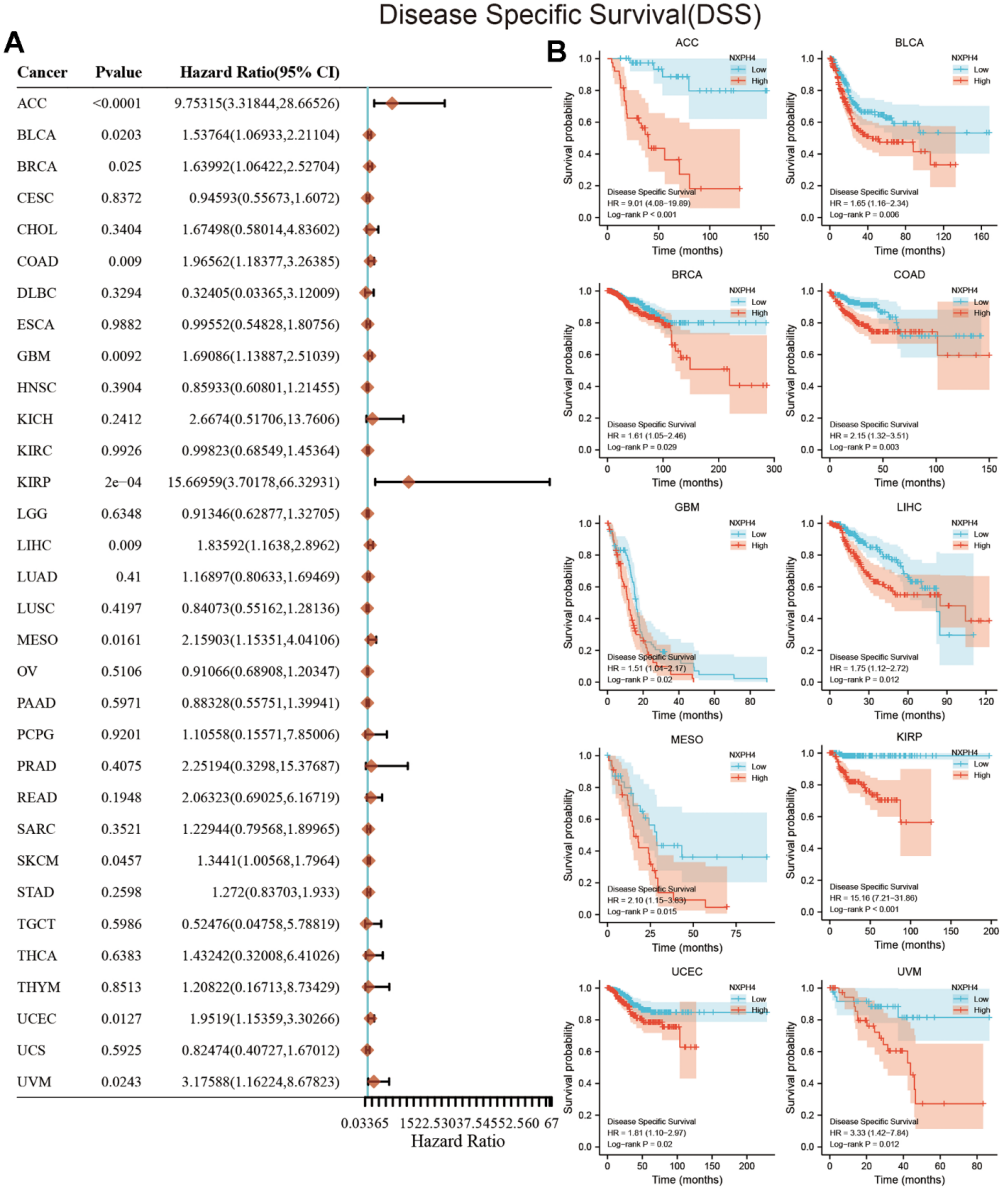
**Relationship between NXPH4 expression and DSS in pan-cancer.** (**A**) Univariate Cox analysis showed the relationship between NXPH4 and DSS in pan-cancer. (**B**) K-M survival analysis showed the relationship between NXPH4 and DSS in pan-cancer.

**Figure 5:**
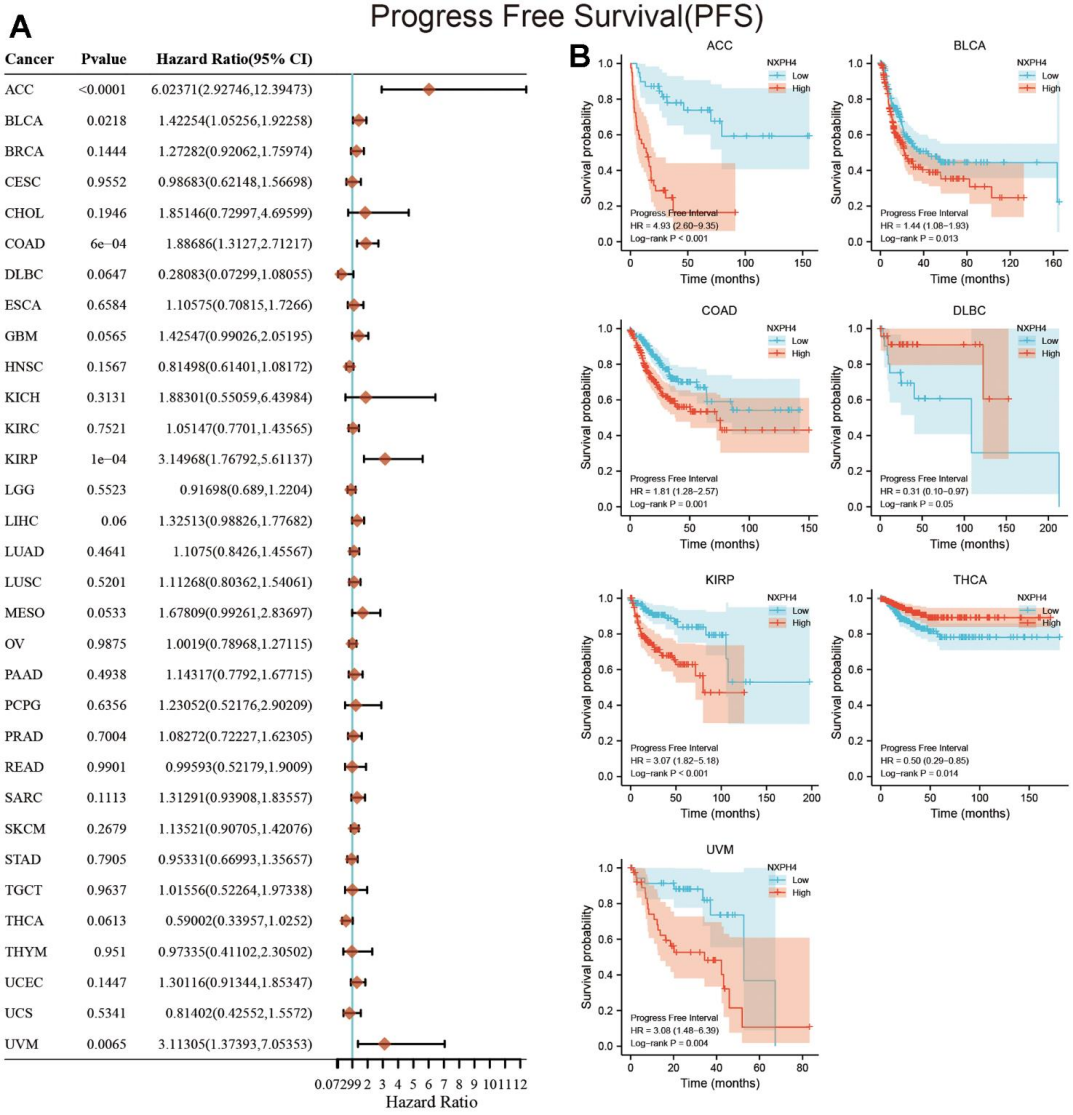
**Relationship between NXPH4 expression and PFS in pan-cancer.** (**A**) Univariate Cox analysis showed the relationship between NXPH4 and PFS in pan-cancer. (**B**) K-M survival analysis showed the relationship between NXPH4 and PFS in pan-cancer.

The current research used univariate Cox regression analysis and KM survival analysis to assess the link between NXPH4 levels and patient survival. At the OS level, the outcomes of the univariate Cox analysis highlighted a link between elevated NXPH4 expression levels and worse OS in ACC, BLCA, BRCA, COAD, Glioblastoma multiforme (GBM), KIRP, LIHC, SKCM, and UCEC (Figure 3A). Patients belonging to the high NXPH4 expression group exhibited poorer OS in ACC, BLCA, BRCA, COAD, GBM, KIRP, GBM, LIHC, READ, UVM, and UCEC (Figure 3B). At the DSS level, univariate Cox analysis indicated a link between elevated NXPH4 levels and worse DSS in ACC, BLCA, COAD, GBM, KIRP, LIHC, MESO, SKCM, and UCEC (Figure 4A). KM survival analysis highlighted that individuals in the NXPH4 high expression group were linked to poorer DSS in ACC, BLCA, BRCA, COAD, GBM, KIRP, GBM, LIHC, MESO, KIRP, UVM, and UCEC (Figure 4B). At PFS, univariate Cox analysis showed higher NXPH4 expression associated with poorer PFS in ACC, BLCA, COAD, and UVM (Figure 5A). According to the KM survival analysis, patients in the high NXPH4 expression group experienced inferior progression-free survival (PFS) outcomes for ACC, BLCA, COAD, DLBC, KIRP, THCA, and UVM, as illustrated in Figure 5B.

### NXPH4 in relation to the immune microenvironment, TMB, and MSI

**Figure 6:**
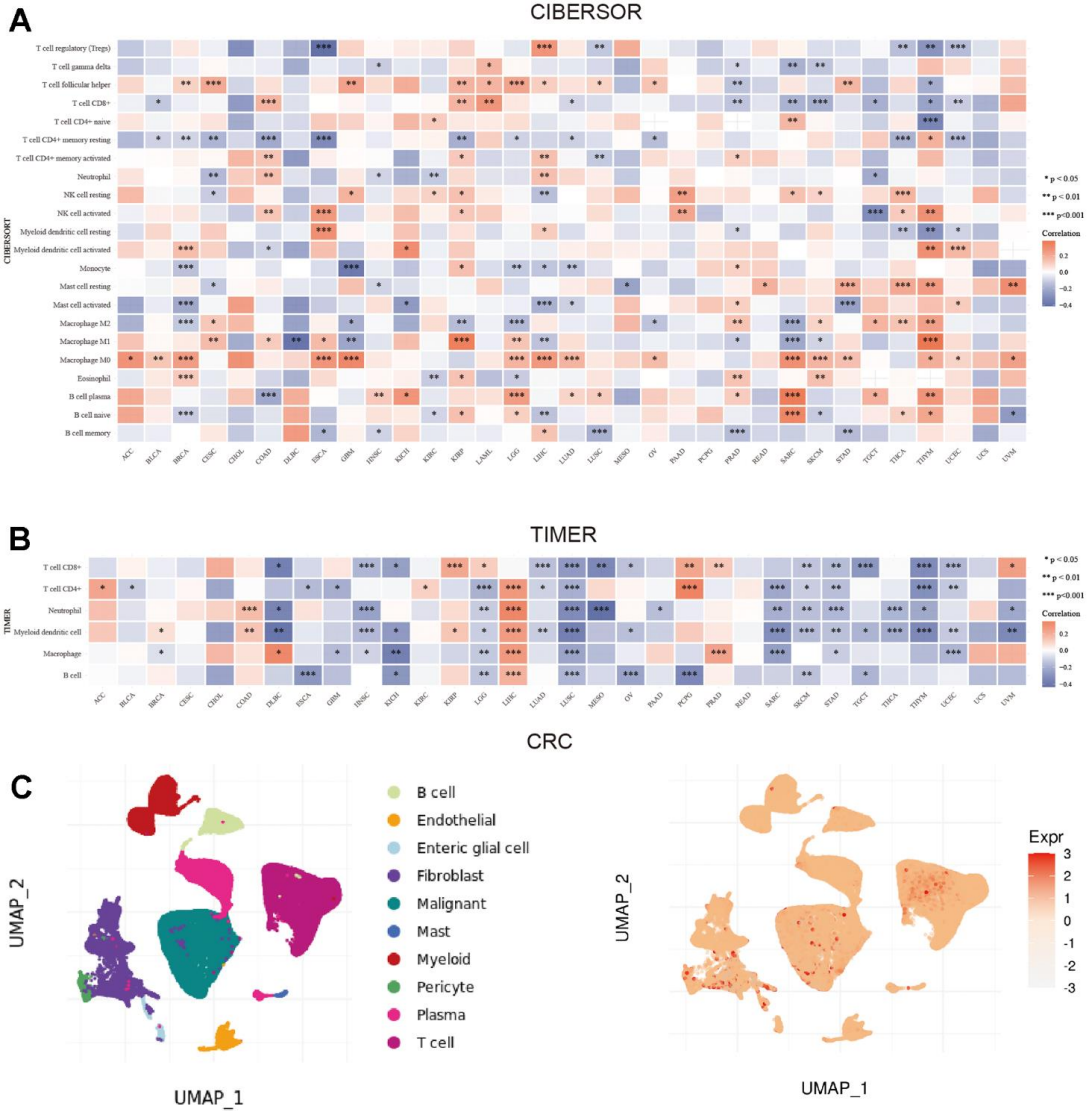
**Correlation between NXPH4 and immune microenvironment in pan-cancer and single cell analysis in colorectal cancer.** (**A**) CIBERSORT algorithm was used to calculate the correlation between NXPH4 expression and immune cell infiltration in pan-cancer. (**B**) Correlation between NXPH4 expression and immune cell infiltration was calculated by TIMER algorithm in pan-cancer. (**C**) The expression of NXPH4 in colorectal cancer at the single-cell level was analyzed based on TIGER database.

The CIBERSORT and TIMER algorithms were utilized to evaluate the link between NXPH4 and the abundance of individual immune cell types across various cancer types (pan-cancer analysis). The outcomes from the CIBERSORT analysis highlighted that NXPH4 demonstrated a positive link to follicular helper T cells, M0 macrophages, and plasma cells while showing a negative correlation with CD8+ T cells, CD4+ memory T cells, and monocytes in most cancers (Figure 6A). TIMER algorithm results showed that NXPH4 exhibited a negative link to CD8+ T-cells, CD4+ T-cells, neutrophils, macrophages, and B-cells in most cancers (Figure 6B). However, in LIHC, NXPH4 exhibited a positive link to CD4+ T cells, neutrophils, macrophages, and B cells, suggesting that NXPH4 may have different immune effects in various cancers (Figure 6B). Subsequently, the current investigation examined the single-cell sequencing data for colorectal cancer published by Lee HO et al. using the TIGER database. The analysis included the identification of subtype clustering in this data as well as the evaluation of NXPH4 expression levels across various cell types, as illustrated in Figure 6C. Moreover, NXPH4 was predominantly expressed in malignant cells and only weakly expressed in other cell types (Figure 6C).

**Figure 7:**
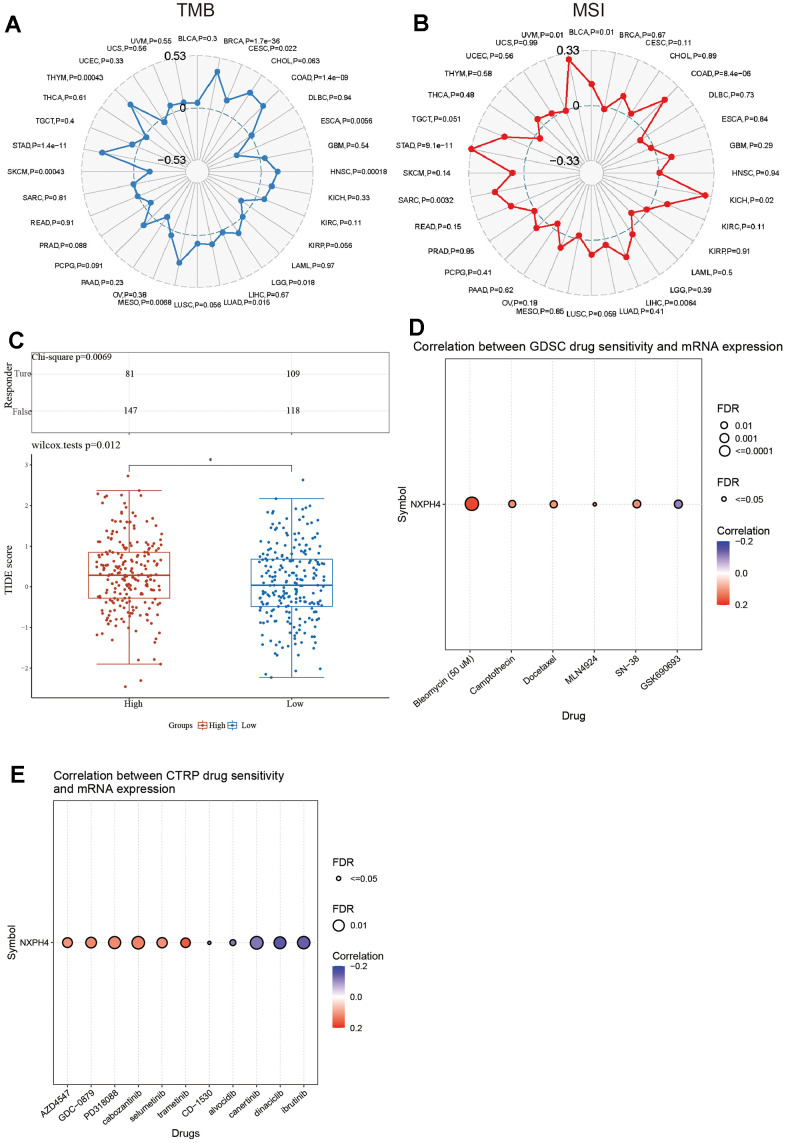
**NXPH4 and drug sensitivity in colon cancer.** (**A**) Relationship between NXPH4 and TMB in colon cancer. (**B**) Relationship between NXPH4 and MSI in colon cancer. (**C**) Difference of TIDE score between high and low NXPH4 expression groups in colon cancer. (**D**) GSCA online tool was used to analyze the correlation between NXPH4 expression and chemotherapeutic drug IC50 in GDSC database. (**E**) GSCA online tool to analyze the correlation between NXPH4 expression and chemotherapy drug IC50 in CTRP database.

Furthermore, NXPH4 exhibited a positive link to TMB in BRCA, CESC, COAD, HNSC, LGG, LUAD, MESO, STAD, and THYM and a negative link to TMB in ESCA and SKCM (Figure 7A, all *P* < 0.05). NXPH4 also showed a positive link to MSI in BLCA, COAD, KIHC, LIHC, SARC, STAD, and UVM (Figure 7B, all *P* < 0.05).

### Link between NXPH4 and sensitivity to drug treatment

The TIDE algorithm was employed to investigate the association of NXPH4 with the responsiveness of COAD patients to immunotherapy. A higher TIDE score suggests lower sensitivity to immunotherapy. COAD patients with elevated NXPH4 expression levels exhibited higher TIDE scores in contrast with those with low NXPH4 expression (Figure 7C). Subsequently, the TIDE score was utilized to predict the immunotherapy response of COAD patients. A higher proportion of COAD patients belonging to the low NXPH4 expression group responded to immunotherapy, suggesting that NXPH4 is associated with poorer sensitivity to immunotherapy (Figure 7C).

Next, the relationship between NXPH4 and chemotherapeutic drug sensitivity was explored using the GSCA database. Analysis based on the GDSC database demonstrated that NXPH4 levels exhibited a positive link to the IC50 values of bleomycin (50 um), camptothecin, docetaxel, MLN4924, and SN-38, while a negative link to the IC50 of GSK690693 (Figure 7D). Furthermore, analysis carried out by utilizing the CTRP database demonstrated a positive link between NXPH4 levels and the IC50 values of several compounds, including AZD4547, GDC-0879, PD318088, cabozantinib, trametinib, CD-1530, alvocidlb, canertinib, dinaciclib and ibrutinib (as shown in Figure 7E).

### PPI network and functional enrichment analysis

**Figure 8:**
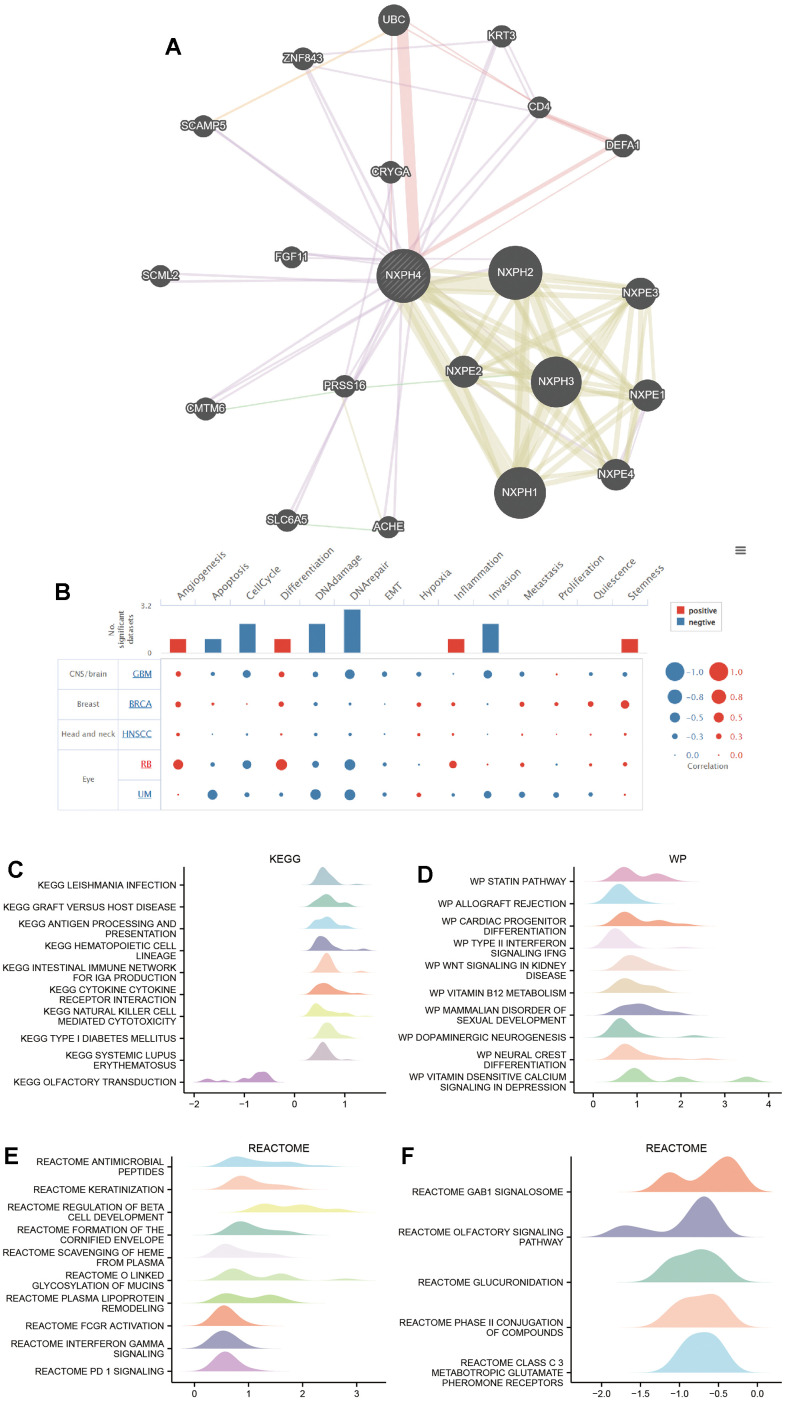
**PPI network and functional enrichment analysis.** (**A**) GeneMANIA database was used to analyze the 20 proteins that NXPH4 interacts with. (**B**) CancerSEA database analysis of NXPH4 in relation to 14 biological processes in various cancers. (**C**) KEGG-based GSEA analysis of NXPH4 in colon cancer. (**D**) Wp-based GSEA analysis of NXPH4 in colon cancer. (**E**, **F**) Reactome-based GSEA analysis of NXPH4 in colon cancer.

For NXPH4 and its associated genes, PPI protein interaction network maps were developed. The major proteins that interact with NXPH4 are the NXPH family (NXPH1, NXPH2, NXPH3) and the NXPE family (NXPE1, NXPE2, NXPE3, NXPE4) (Figure 8A).

Subsequently, a functional enrichment analysis of NXPH4 in each cancer at the single-cell level was performed using the CancerSEA database. In GBM, BRCA, HNSVC, Retinoblastoma (RB), and Uveal melanoma (UM), high NXPH4 expression was mainly positively linked to Angiogenesis and Differentiation and negatively linked to DNA damage, DNA repair, EMT, apoptosis, cell cycle, and invasion (Figure 8B). Figure 8B shows that there was a negative link between most of the biological activities observed in both GBM and UM and elevated levels of NXPH4.

In order to examine the biological role of NXPH4 in colorectal cancer, a GSEA enrichment analysis was carried out. The KEGG pathway-based gene enrichment analysis demonstrated that NXPH4 expression exhibited a positive link to LEISHMANIA INFECTION, GRAFT VERSUS HOST DISEASE, ANTIGENE PROCESSING AND PRESENTATION, HAEMATOPOIETIC CELL LINEAGE, INTESTINAL IMMUNE NETWORK FOR IGA PRODUCTION, CYTOKINE CYTOKINE RECEPTOR INTERACTION, NATURAL KILLER CELL-MEDIATED CYTOTOXICITY, TYPE I DIABETES MELLITUS, SYSTEMIC LUPUS ERYTHEMATOSUS. On the other hand, NXPH4 expression exhibited a negative link to olfactory transduction (Figure 8C). According to the gene enrichment analysis based on the WP database, the upregulation of NXPH4 expression was found to be positively linked to STATIN PATHWAY, ALLOGRAFT REJECTION, CARDIAC PROGENITOR, DIFFERENTIATION, TYPE II INTERFERON-SIGNALING IFNG, WNT SIGNALING IN KIDNEY DISEASE, VITAMIN B12 METABOLISM, MAMMALIAN DISORDER OF SEXUAL DEVELOPMENT, DOPAMINERGIC NEURO-GENESIS, NEURAL CREST DIFFERENTIATION, and VITAMIN SENSITIVE CALCIUM SIGNALING IN DEPRESSION (as depicted in Figure 8D). Based on the gene enrichment analysis conducted using the REACTOME database, it was observed that NXPH4 exhibited positive associations with several biological processes. These processes include antimicrobial peptides, keratinization, regulation of beta cell development, formation of the cornified envelope, scavenging of heme from plasma, O-linked glycosylation of mucins, plasma lipoprotein remodeling, Fcgr action, interferon-gamma signaling, and PD1 signaling (Figure 8E). Additionally, the analysis also highlighted positive correlations between NXPH4 and GAB1 signalosome, olfactory signaling pathway, glucuronidation, phase II conjugation of compounds, and class C 3 metabotropic glutamate pheromone receptors (Figure 8F).

### sh-NXPH4 suppresses HT29 and HCT116 cell proliferation, migration, and invasion

**Figure 9:**
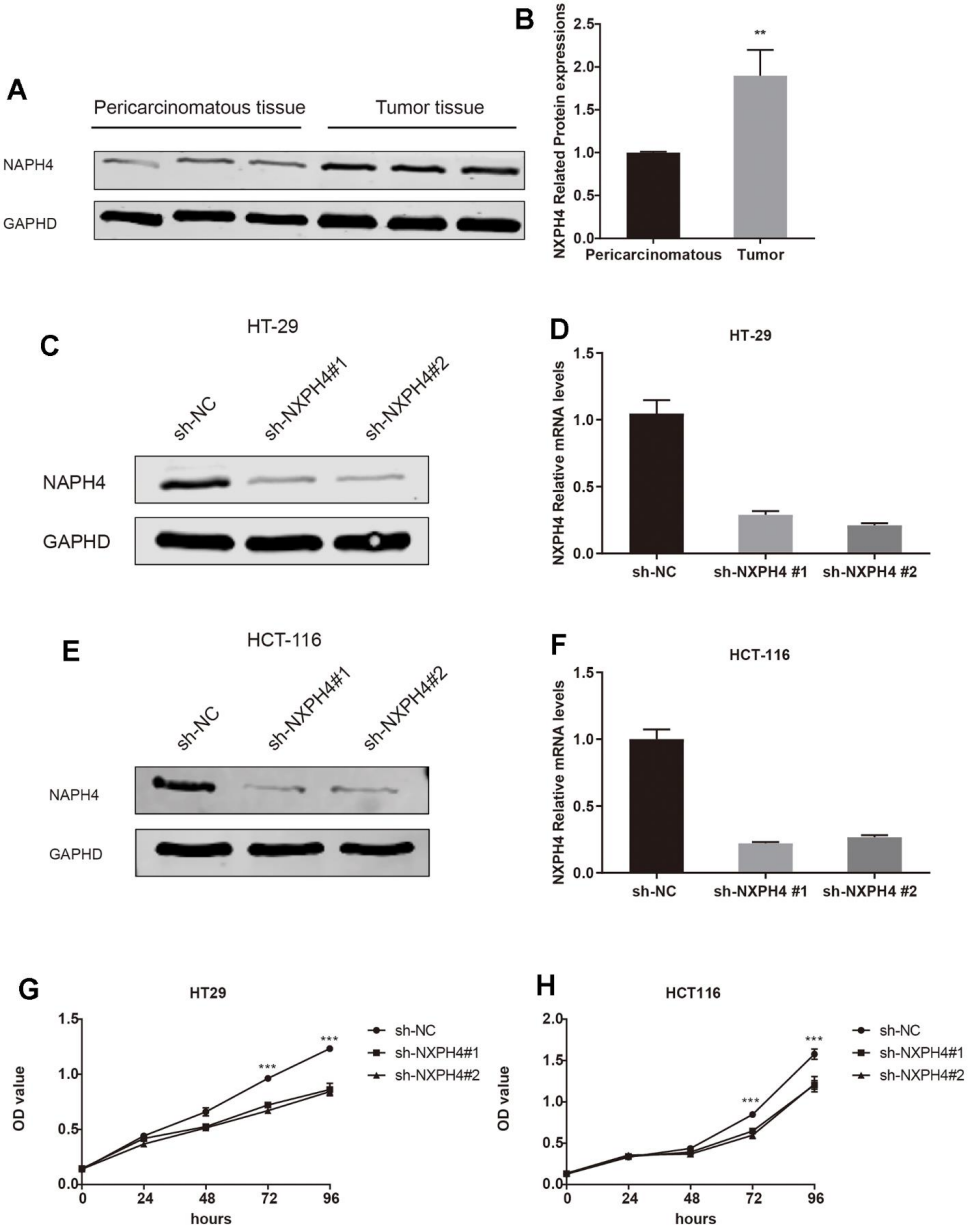
**The expression of NXPH4 in colon cancer tissues was verified and knocked down in cell lines.** (**A**, **B**) Western blot was used to verify the expression of NXPH4 at the protein level in colon cancer and para-cancer tissues. (**C**) NXPH4 was knocked down in HT-29 cell line and verified by Western blot. (**D**) NXPH4 was knocked down in HT-29 cell line and verified by qPCR. (**E**) NXPH4 was knocked down in HCT-116 cell line and verified by Western blot. (**F**) NXPH4 was knocked down in HCT-116 cell line and verified by qPCR. (**G**) CCK8 assay of control HT-29 cell line and HT-29 cell line with NXPH4 knockdown. (**H**) CCK8 assay for control HCT-116 cell line and HCT-116 cell line with NXPH4 knockdown.

Firstly, the current research observed the presence of NXPH4 protein in samples obtained from patients with colorectal cancer as well as from healthy individuals. The findings indicated a substantial increase in NXPH4 protein expression levels within colorectal cancer tissues, which was consistent with previous research outcomes (Figure 9A, 9B). Subsequently, colorectal cancer cell lines HT29 and HCT116 were transfected with sh-NC and sh-NXPH4 (Figure 9C–9F), and their impact on cell proliferation, migration, and invasion was assessed. Through the CCK8 assay, it was observed that sh-NXPH4 had a remarkable inhibitory effect on the proliferation of colorectal cancer cell lines HT29 and HCT116 (Figure 9G, 9H). The plate clonogenicity assay yielded similar findings, with a remarkable reduction in the number of cell colonies observed in the sh-NXPH4 group compared to the sh-NC group (Figure 10A–10D). The Transwell assay outcomes demonstrated significant suppression of the invasion capability in colorectal cancer cell lines HT29 and HCT116 for the sh-NXPH4 group when compared to the sh-NC group (Figure 10E–10H). By conducting a wound healing assay, it was observed that sh-NXPH4 had a considerable inhibitory impact on the migration ability of colorectal cancer cell lines HT29 and HCT116 (Figure 10I–10K). These findings revealed that the knockdown of NXPH4 expression resulted in significant suppression of the proliferation, migration, and invasion of colorectal cancer cell lines HT29 and HCT116.

**Figure 10:**
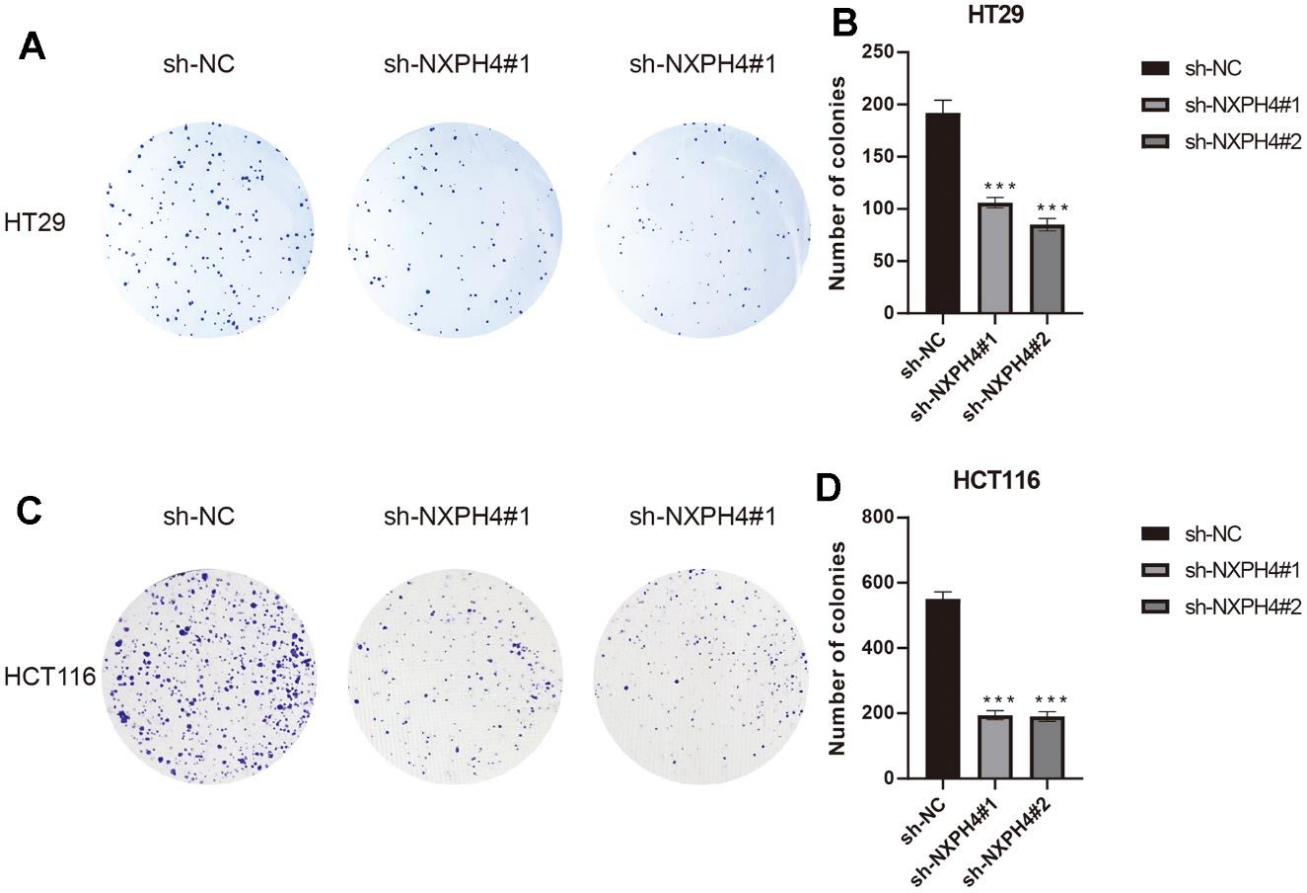
**Effects of NXPH4 knockdown on proliferation, invasion and migration of HT-29 and HCT-116 cell lines.** (**A**, **B**) Plate clonogenicity assay showed the effect of NXPH4 knockdown on the proliferative ability of HT-29 cell line. (**C**, **D**) Plate clonogenicity assay showed the effect of NXPH4 knockdown on the proliferative ability of HCT-116 cell line.

**Figure 10:**
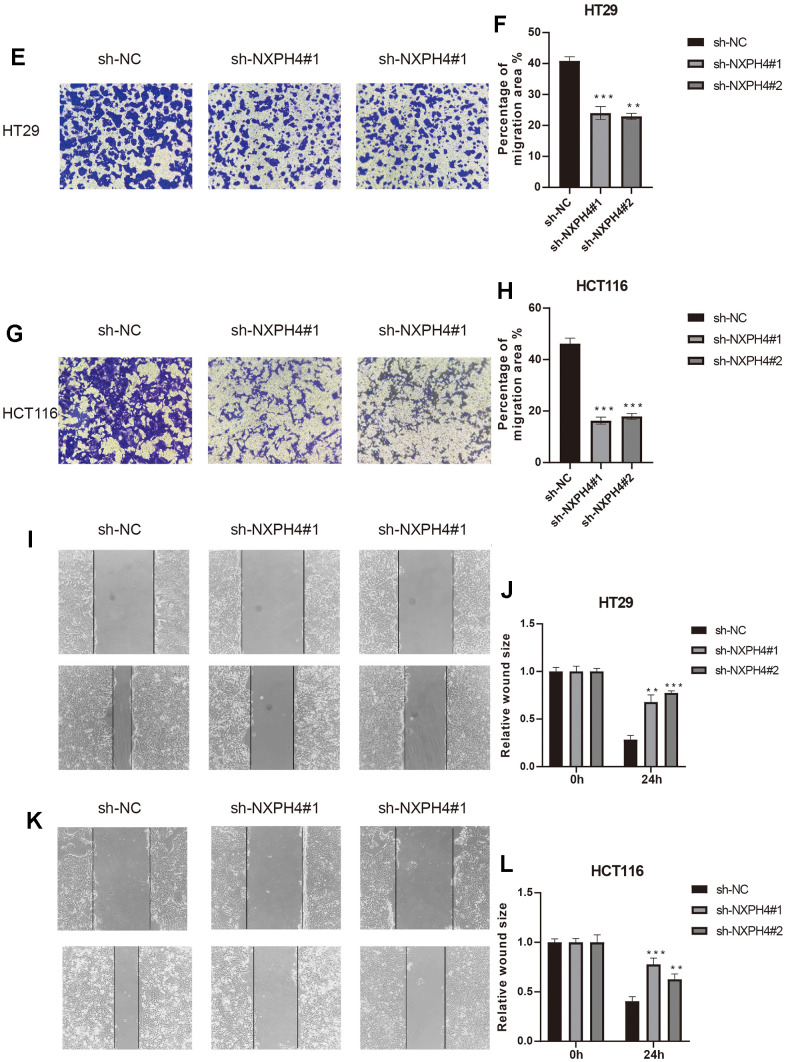
**Effects of NXPH4 knockdown on proliferation, invasion and migration of HT-29 and HCT-116 cell lines.** (**E**, **F**) Transwell assay showed the effect of knocking down NXPH4 on the invasion ability of HT-29 cell line. (**G**, **H**) Transwell assay showed the effect of NXPH4 knockdown on the invasion ability of HCT-116 cell line. (**I**, **J**) Wound healing assay showed the effect of NXPH4 knockdown on the invasion ability of HT-29 cell lines. (**K**, **L**) Wound healing assay showed the effect of NXPH4 knockdown on the invasion ability of HCT-116 cell line.

## DISCUSSION

This study investigated the expression level, diagnostic value, copy number variation, methylation, prognostic value, and immunological relevance of NXPH4 on pan-cancer. NXPH4 is upregulated in most cancers and has a high diagnostic value. Dysregulation of NXPH4 expression in some cancers may be associated with abnormal copy number variation and methylation. In addition, NXPH4 is associated with poorer prognosis in many cancers and could be used as a prognostic marker. Importantly, functional studies have shown that NXPH4 can promote colon cancer progression.

The role of NXPH4 in cancer has only recently been investigated. NXPH4 shows a higher expression level in LIHC, BLCA, and non-small cell lung cancer [16, 17, 27], but the cause of NXPH4 dysregulation remains unclear. Our study also revealed a higher NXPH4 expression level in the transcriptome of most cancers. In addition, the current research analyzed the methylation levels and copy number variation of NXPH4 in an attempt to explain the possible mechanisms of its dysregulation in some cancers. It was found that NXPH4 amplification mutations were more frequent in LUAD, HNSC, OV, PAAD, COAD, LIHC, LGG, USC, BLCA, PRAD, SKCM, STAD, SARC, and KIRC and that NXPH4 expression was proportional to the copy number. The methylation levels of NXPH4 in BLCA, COAD, KIRC, KIRP, LIHC, PRAD, THCA, and UCEC were lower than in the corresponding paracancerous tissues, and hypomethylation of the gene was a cause of the high expression in these tumors [28, 29]. These findings indicate that the underlying causes of NXPH4 dysregulation vary across different types of cancer. Therefore, additional experiments are required to identify the mechanisms responsible for its abnormal expression in cancer.

Jung et al. also found that serum NXPH4 protein has diagnostic ability in LIHC [18]. Our study also found that NXPH4 has the potential to differentiate between cancerous and paraneoplastic tissues in 12 types of cancers, including LIHC (Figure 1C). However, further studies in peripheral blood are needed to translate the diagnostic potential of NXPH4 into clinical application, and NXPH4 also has the potential to serve as a prognostic marker. In BLCA and LIHC, NXPH4 was associated with a worse prognosis [17, 27]. The findings of this research validate previous research and analyze the link between NXPH4 and OS, DSS, and PFS in pan-cancer. In a recent study, Chen et al. systematically analyzed single-cell transcriptome data and Bulk transcriptome data of colorectal cancer to construct a stem cell-related prognostic model, with NXPH4 being one of the elements of this model, indicating the potential of NXPH4 in predicting the prognosis of colorectal cancer [30]. Our study specifically investigated the association between NXPH4 gene expression and the prognosis of colon cancer. Results from K-M survival analysis and Cox analysis both indicated that NXPH4 is associated with a poorer prognosis in COAD patients. Although NXPH4 has excellent potential in cancer diagnosis and prognosis, its molecular function is still poorly understood. In BLCA, NXPH4 promoted cancer cell proliferation and metastasis *in vitro* and induced gemcitabine resistance in BLCA, partly by enhancing glycolytic activation [12]. Knockdown of NXPH4 in LIHC cell lines by Tang et al. also resulted in reduced proliferation and invasive capacity of cancer cells [12]. In Non-small Cell Lung Cancer (NSCLC), NXPH4 is regulated by its upstream transcription factor E2H2, which promotes cancer cell proliferation and invasion by altering the structure of CDKN2A [16]. The current research further knocked down NXPH4 in the COAD cell lines HCT116 and HT29 and found that the proliferation and invasion ability of HCT116 and HT29 were downregulated after NXPH4 knockdown. Our findings, together with the previous studies, suggest that NXPH4 acts as an oncogene to promote cancer development in various settings.

There is increasing evidence that innate immune cells (e.g., macrophages, NK cells, and neutrophils) and adaptive immune cells (including B cells and T cells) can shape the tumor microenvironment and promote tumor growth, metastasis, and drug resistance [31–33]. This role of the tumor microenvironment has also been confirmed in COAD [34, 35]. By correlating NXPH4 with immune cells in the tumor microenvironment, it was observed that NXPH4 was linked to different immune cell infiltrations in various cancers. In most cancers, NXPH4 exhibited a positive link to follicular helper T cells, M0 macrophages, and plasma cells, and a negative link to CD8+ T cells, CD4+ memory T cells, and monocytes. CD8+ T cells are the main effector cells of anti-tumor immunity. They work by recognizing and killing tumor cells to resist the growth and spread of tumors. CD8+ T cells identify tumor cell surface antigens and release cytotoxins such as perforin and interferon to directly destroy tumor cells. Additionally, CD8+ T cells can also activate other immune cells, such as natural killer cells and macrophages, to enhance their ability to attack tumors [36, 37]. This suggests that NXPH4 may be associated with the immunosuppressive tumor microenvironment (TME); however, there are currently no reports on the impact of NXPH4 on the TME of tumors, and detailed mechanisms still require further functional experiments to be determined.

In recent years, immunotherapy has emerged as a groundbreaking approach to treating cancer. Nevertheless, the effectiveness of immunotherapy varies, as it produces positive outcomes in only a proportion of patients. A significant proportion of patients remain unresponsive to immunotherapy, and this resistance is particularly evident in colorectal cancer [38]. The current study used the TIDE algorithm to predict the sensitivity of colorectal cancer patients to immunotherapy and found that patients with high NXPH4 expression had higher TIDE scores, which implies less sensitivity to immunotherapy [23]. This may be attributed to the positive correlation between NXPH4 and MSI and TMB in COAD. MSI-high (MSI-H) tumors have characteristics such as high immunogenicity, strong lymphocyte infiltration in the tumor microenvironment, good prognosis, and insensitivity to conventional chemotherapy and radiotherapy, making them the main beneficiaries of immunotherapy [39, 40]. TMB is another indicator for predicting the sensitivity of COAD to immunotherapy. It has been reported that colorectal cancer patients with high TMB (TMB≥8 muts/Mb) have longer OS compared to those with low TMB [41]. Furthermore, TMB has been reported as another predictive biomarker for MSI in metastatic colorectal cancer. In MSI-H mCRC patients, those with high TMB (TMB cutoff defined as 37-41 mutations/Mb) have a better prognosis after receiving immunotherapy than those with low TMB [42]. In the Canadian Cancer Trials Group CO.26 study, MSS colorectal cancer patients with elevated plasma TMB levels (≥28 muts/Mb) showed predictable responses to the combination therapy of the anti-PD-L1 drug durvalumab and the anti-CTLA4 drug tremelimumab [43]. Thus, NXPH4 expression might partially indicate the immune status of the tumor microenvironment and predict the sensitivity of colorectal cancer patients to immunotherapy.

## CONCLUSIONS

In conclusion, this study revealed the dysregulation of NXPH4 at a pan-cancer level and suggested the potential of NXPH4 as a diagnostic and prognostic biomarker for certain cancer. NXPH4 can serve as a diagnostic, prognostic, and immunotherapeutic marker, and may promote the proliferation and metastasis of COAD.
